# Investigation of the inhibition effect and mechanism of myricetin to Suilysin by molecular modeling

**DOI:** 10.1038/s41598-017-12168-y

**Published:** 2017-09-18

**Authors:** Xiaodi Niu, Lin Sun, Guizhen Wang, Yawen Gao, Yanan Yang, Xiyan Wang, Hongsu Wang

**Affiliations:** College of food science and engineering, Jilin University, Changchun, China

## Abstract

In the present study, the inhibitory effect and mechanism of myricetin, a natural flavonoid compound, in relation to Suilysin (SLY) were investigated through molecular dynamics simulations, mutational analysis and fluorescence-quenching assays. Myricetin is a potential inhibitor that does not exhibit antimicrobial activity but has been shown to inhibit SLY cytotoxicity. Molecular dynamics simulations and mutational analysis revealed that myricetin binds directly to SLY in the gap between domains 2 and 3, an important region for oligomerization and pore formation. The results of principal component analysis (PCA) indicated that the binding of myricetin in this gap region restricts the conformational transition of SLY from a monomer to an oligomer, thereby counteracting the haemolytic activity of SLY. This mechanism was verified using a haemolysis assay. These results demonstrated that myricetin is a strong candidate as a novel therapeutic agent for the treatment of *Streptococcus suis* infections.

## Introduction

Among the bacterial pore-forming toxins, the cholesterol-dependent cytolysins (CDCs) comprise the largest family. The CDC Suilysin (SLY) is an essential virulence factor of *Streptococcus suis*. This Gram-positive pathogen secretes the water-soluble monomers of SLY, which binds to and subsequently forms pores in the membranes of host cells^1,2^.

To explore the function of SLY, the 3D structure of the SLY monomer was solved using X-ray crystallography^3^. According to previous reports, SLY comprises four distinct domains, referred to as domains 1 to 4. With the exception of domain 4, the other three domains are intertwined, and more importantly, domain 4 can promote the initial binding of SLY with cholesterol-containing membranes^4–6^. During the self-assembly of the pores in the membranes of host cells, SLY monomer molecules scatter in all directions to form a prepore complex^7^. Then, two *β*-hairpins contribute to the formation an oligomer *β*-barrel pore comprising each SLY monomer^8,9^. A total of 30–50 SLY monomers are involved in the formation of an oligomer pore with a diameter of 250–350 Å^10,11^. A subsequent conformational change in the SLY monomers occurs with pore formation, in which two helix bundles in each monomer are converted into a pair of amphiphilic transmembrane *β*-hairpins that insert into the membrane^9,12,13^.

Many studies have shown that SLY exerts an important influence on the pathogenesis of *Streptococcus suis* ^14–16^, and analysis of the effects of SLY on the host inflammatory response can help to elucidate the roles of SLY during the regulation of the signalling pathways responsible for the severity of STSLS and meningitis^17^. In mucosal infections of complement-deficient mice with *Streptococcus suis*, SLY and the capsule play critical roles^18^. It has been shown that SLY expression facilitates early disease onset and the pathogenesis of meningitis in experimentally infected mice carrying an SLY mutation. In addition, SLY stimulates the release of heparin-binding protein from polymorphonuclear neutrophils and mediates vascular leakage in mouse infection models as a result of calcium influx-dependent degranulation^19^. Furthermore, it has been confirmed that SLY can contribute to the development of bacterial meningitis and the resulting increase in mortality based on the work of Dan Takeuchi *et al*.^20^.

Considering the crucial role of SLY in the pathogenicity of *Streptococcus suis*, SLY may be a potential drug target in infections caused by this bacterium. Unfortunately, there are few related reports of SLY inhibitors. Therefore, studies aimed at discovering novel potent inhibitors of SLY and additional research to confirm the mechanism of interaction between SLY and its inhibitors are essential. In our previous literature, it was reported that morin, a natural compound could inhibit the haemolytic activity of SLY by restraining the transformation from the monomer form to the oligomer form based on the binding direct to the domain 2 of SLY^21^. In the present study, we observed that the natural compound myricetin (MYR) could inhibit the haemolytic activity of SLY. Furthermore, the mechanism of SLY inhibition by MYR was determined through molecular modelling, free energy calculations, and haemolysis release assays. It was verified that MYR binds directly to the gap between domains 2 and 3 in SLY, and amino acid residues Asn82, Ile87, Lys192, and Phe193 play key roles in the binding of MYR with SLY. Principal component analysis (PCA) revealed that the binding of MYR in the gap region restricted the conformational transition of SLY from a monomer to an oligomer, thereby counteracting the haemolytic activity of SLY. This mechanism was verified using a haemolysis assay.

## Results and Discussion

### MYR inhibits the haemolytic activity of SLY

**Figure 1:**
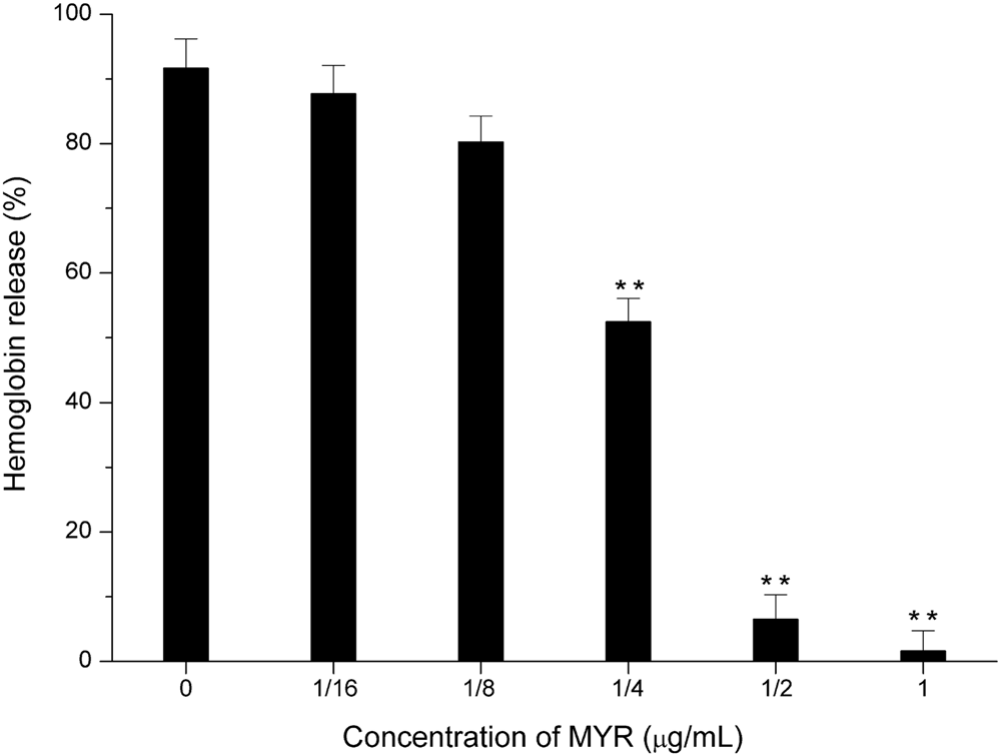
The inhibitory effect of MYR on SLY-induced haemolysis. The results of haemolysis tests using purified rSLY and sheep blood cells to measure the absorbance values of the centrifugal supernatants at 543 nm confirmed that the SLY-induced haemolysis could be reduced by MYR. The column diagrams show the average values for the assays (*n* = 3). **Compared with the matched group, *P* < 0.01.

In the present study, the natural compound MYR, which does not exhibit anti-bacterial activity, attenuated the haemolytic activity of SLY when mixed with purified SLY, indicating that MYR directly interacts with the SLY protein (Fig. 1).

### Determination of the binding mode of SLY with MYR

**Figure 2:**
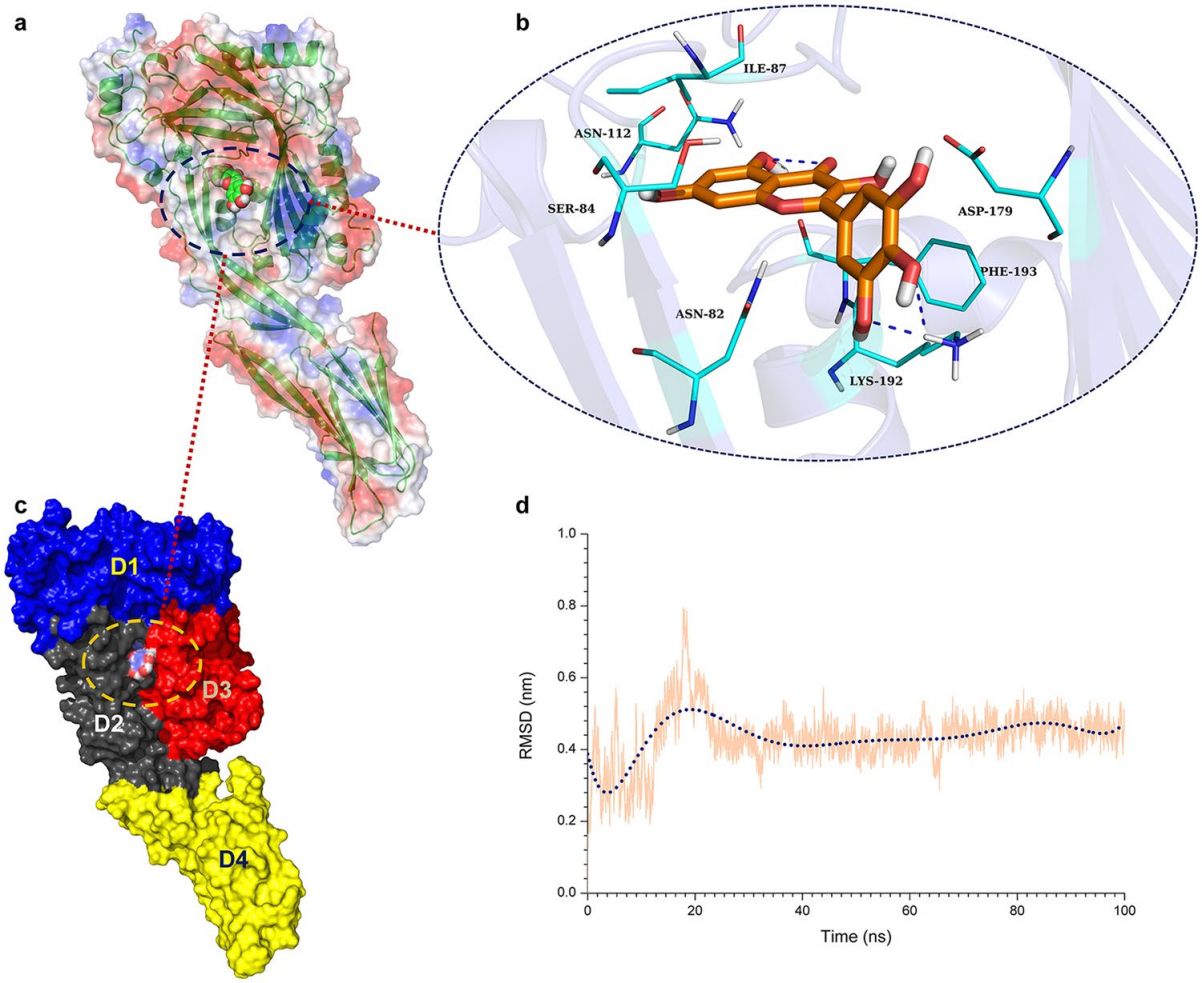
The binding mode of MYR with SLY based on molecular dynamics simulation. (**a**), (**c**) MYR can bind to the gap region between D2 and D3 in SLY; (**b**) residues of the binding site in the SLY-MYR complex; (**d**) the RMSD displayed by the backbone atoms of the protein during MD simulations of the SLY-MYR complex.

The direct interaction of SLY with MYR suggested that the inhibition mechanism of MYR against SLY was important. To explore the binding mode of SLY with MYR, a 100-ns molecular dynamics simulation was performed for the SLY-MYR complex based on the docking results. Initially, the equilibrium of the complex system was verified based on analysis of the root-mean-square deviations (RMSD) of backbone C_α_ atoms. As shown in Fig. 2d, the complex reached equilibrium between 0.4 and 0.6 nm after ~40 ns, verifying that the final 60 ns of the simulation was suitable for analysis.

**Figure 3:**
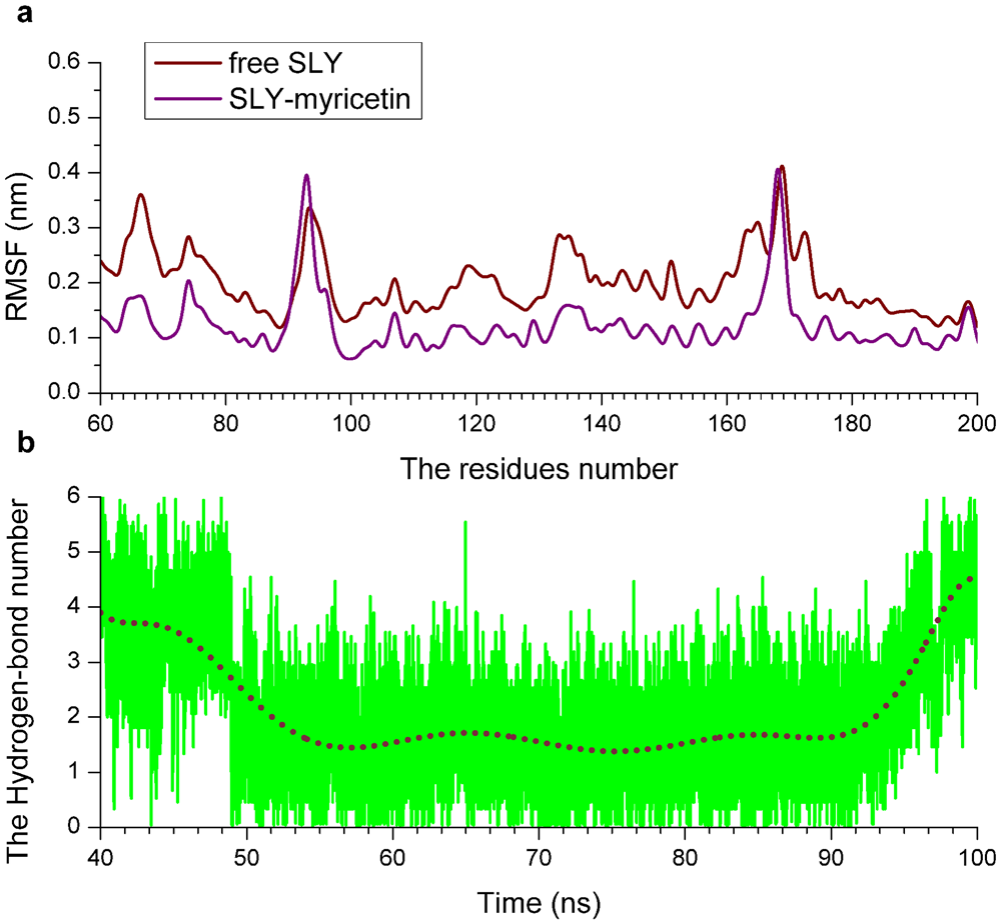
The RMSF of SLY in the complex and the number of hydrogen bonds between MYR and SLY. (**a**) RMSF of the residues over the last 60 ns of the simulation with respect to their initial positions in the free protein and complex system; (**b**) the number of hydrogen bonds between MYR and SLY during the last 60-ns simulation.

Over the time course of the 100-ns simulation, MYR is a ligand that binds to the gap region between D2 and D3 in SLY via Van der Waals and electrostatic interactions. The predicted binding mode of MYR with SLY is shown in Fig. 2a,b and c. In detail, observation of the binding mode of MYR with SLY revealed that amino acid residues Asn82, Asp179, Lys192 and Phe193 could form strong interactions with the benzene ring of MYR. In addition, the side chains of Ser84, Ile87, Asn112 and Phe193 are close to the 4H-chromen-4-one moiety of MYR, indicating that these two residues play key roles in stabilizing MYR. Due to the binding of MYR with residues at the binding sites of SLY the flexibility of these residues in the complex system is different from that in the free protein. As shown in Fig. 3a, the residues at the binding sites (80–90, 170–200) of the complex system showed a low degree of flexibility, with an root mean square fluctuation (RMSF) of less than 0.30 nm, compared with free SLY, indicating that these residues are more rigid as a result of binding to MYR. Moreover, the number of hydrogen bonds was calculated during the simulation from 40 to 100 ns. Figure 3b shows that the number of hydrogen bonds fluctuates between 1 and 3 within the simulation, indicating that there are two hydrogen bonds between MYR and the SLY protein. The relevant information concerning the stability of the hydrogen bonds between MYR and SLY is provided in Table 1.

**Table 1:** SLY-MYR H-bonds from MD simulations.

Acceptor	Donor	Presence %	Distance (Å)
MYR:			
Lig-O	Lys192 N-H	75.8	2.2 ± 0.14
Lig-O		69.7	1.9 ± 0.13

### Identification of the binding sites between MYR and SLY

The sites of MYR binding to SLY were verified using the MM-PBSA method to calculate the interaction decomposition of the binding energy between MYR and each residue of SLY.

**Figure 4:**
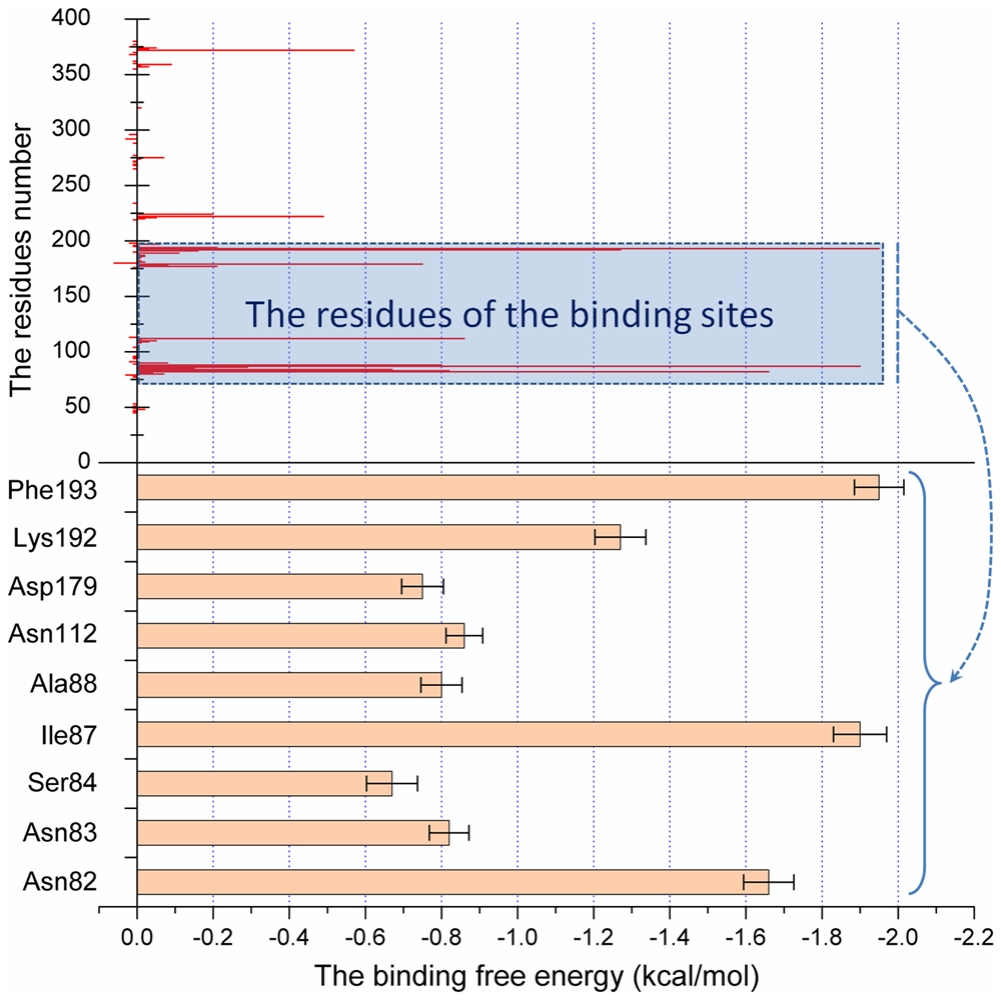
Decomposition of the binding energy of each residue in the SLY-MYR complex. The calculated decomposition free energy showed that the major contribution to the free energy came from Asn82, Ile87, Asp179, Lys192, and Phe193, suggesting that these five residues are key residues for the binding of MYR with SLY.

As shown in Fig. 4, the residues (75–200) in the SLY-MYR binding region exhibited the highest binding free energy (*ΔE*_*total*_ < −0.6 kcal/mol). In detail, Phe193 and Ile87 showed the strongest binding energy with MYR, with a *ΔE*_*total*_ < −1.8 kcal/mol. Combined with the results of the analysis shown in Fig. 2b, Ile87 is sufficiently close to the 4*H*-chromen-4-one moiety of MYR to cause a stronger interaction between MYR and SLY. Moreover, Asn82 exhibits a strong attraction interaction with MYR, with a *ΔE*_*total*_ < −1.2 kcal/mol, indicating that the 4*H*-chromen-4-one moiety of MYR can also be anchored via Asn82. Furthermore, due to the formation of two hydrogen bonds with the benzene ring moiety of MYR (Fig. 2b), strong binding energy of Lys192 with MYR was observed, with a *ΔE*_*total*_ < −1.2 kcal/mol. Asp179 and Asn112 exhibited relatively lower binding energy, with a *ΔE*_*total*_ < −0.8 kcal/mol. As shown in Fig. 2b, Asp179, Lys192 and Phe193 are the only three residues that can interact with the benzene ring moiety of MYR, suggesting that the major contribution to the binding energy between MYR and SLY originates from the 4*H*-chromen-4-one moiety of MYR, while the benzene ring moiety of MYR plays a weak role in the complex system. Based on the calculated decomposition free energy, the major contribution to the free energy came from Asn82, Ile87, Asn112, Asp179, Lys192, and Phe193, suggesting that these six residues are key residues in the binding of MYR with SLY.

To examine this hypothesis, a similar molecular modelling procedure was applied to the complexes of N82A, S84A, N112A and D179A bound with MYR, and the total binding free energies of wild-type SLY (WT- SLY) and SLY mutants with the MYR system were subsequently calculated using the MM-PBSA method. In addition, the binding energies of MYR with WT-SLY and the SLY mutants were measured via a fluorescence-quenching assay. The relevant results are summarized in Table 2. As shown in Table 2, the calculated free energy of binding with MYR was lower for the SLY mutants than for WT-SLY. Interestingly, the experimental results also showed that the binding free energies of the complex systems decrease in a similar order: WT > N82A > N112A > S84A > D179A, consistent with the results of the theoretical simulation. Thus, molecular modelling generated a reliable SLY-MYR complex structure.

**Table 2:** The binding free energy (kcal/mol) of WT-MYR, N82A, S84A, N112A-MYR, and D179-MYR systems based on computational method and the values of the binding constants (*K*_*A*_) based on the fluorescence spectroscopy quenching.

	WT-SLY	N82A	S84A	N112A	D179A
computational method	−14.87 ± 1.24	−11.26 ± 1.16	−9.97 ± 1.04	−10.54 ± 1.01	−8.54 ± 1.01
*K*_A_ (1 × 10^4^) L·mol^−1^	7.39 ± 0.80	6.82 ± 0.91	5.12 ± 0.75	5.78 ± 0.91	−3.85 ± 0.81

### Analysis of the inhibition mechanism through principal component analysis (PCA)

As we all know, principle component analysis (PCA) can be used to address the collective motions of protein based on the positional covariance matrix *C* of the atomic coordinates. Some other similar approaches also can be used to predict the motion of protein, such as the so-called profile-based protein representation, iPro54-PseKNC and iDNA-Prot^22–24^.

**Figure 5:**
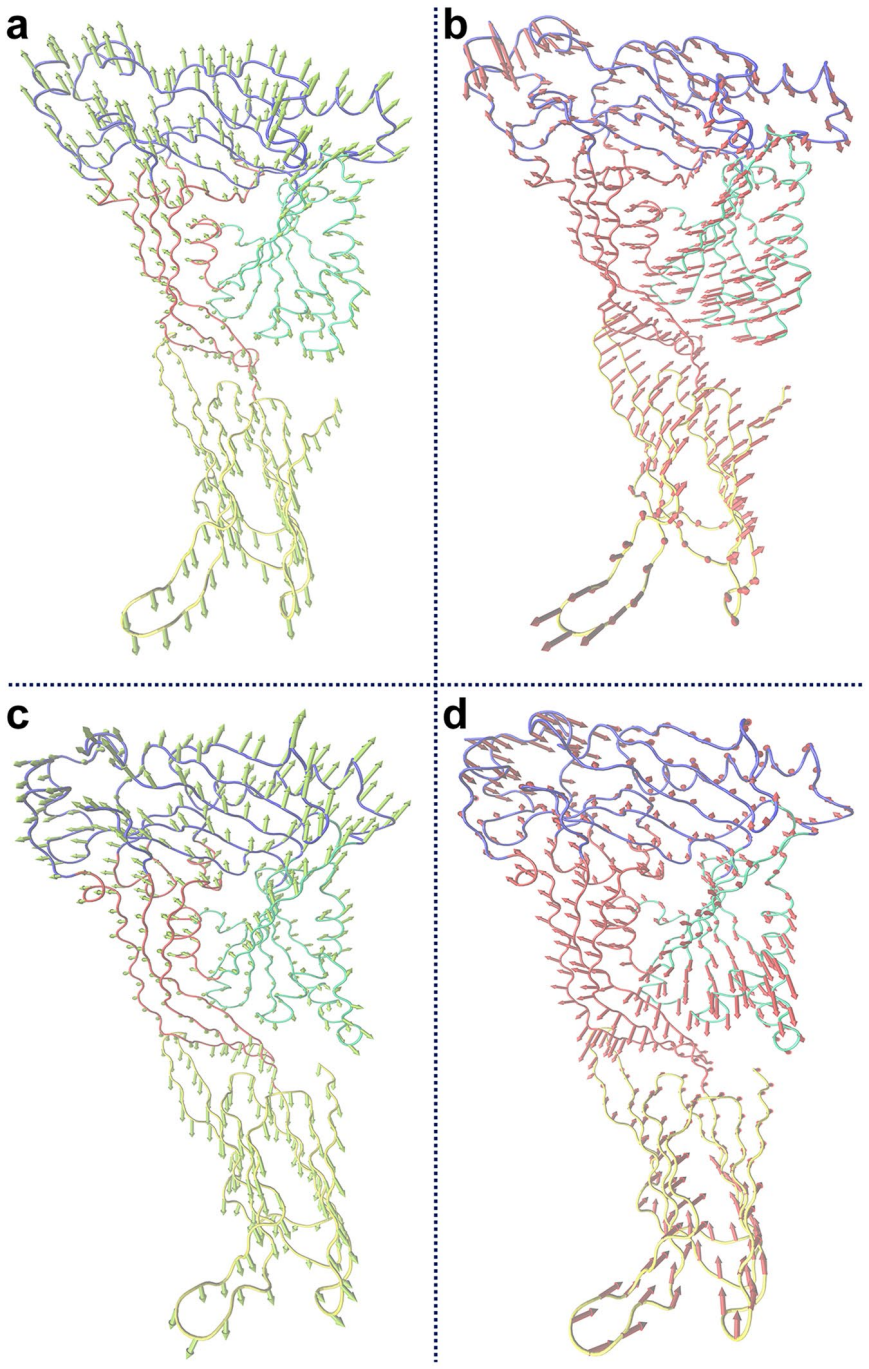
Principal component analysis based on the simulation trajectory. The first (**a**) and second (**b**) principal components (PC1 and PC2) of free SLY obtained through PCA are depicted as cones on the C_α_. The first (**c**) and second (**d**) principal components (PC1 and PC2) in the SLY-MYR complex obtained via PCA are depicted as cones on the C_α_. The length of the cones represents the magnitude of the motion.

**Figure 6:**
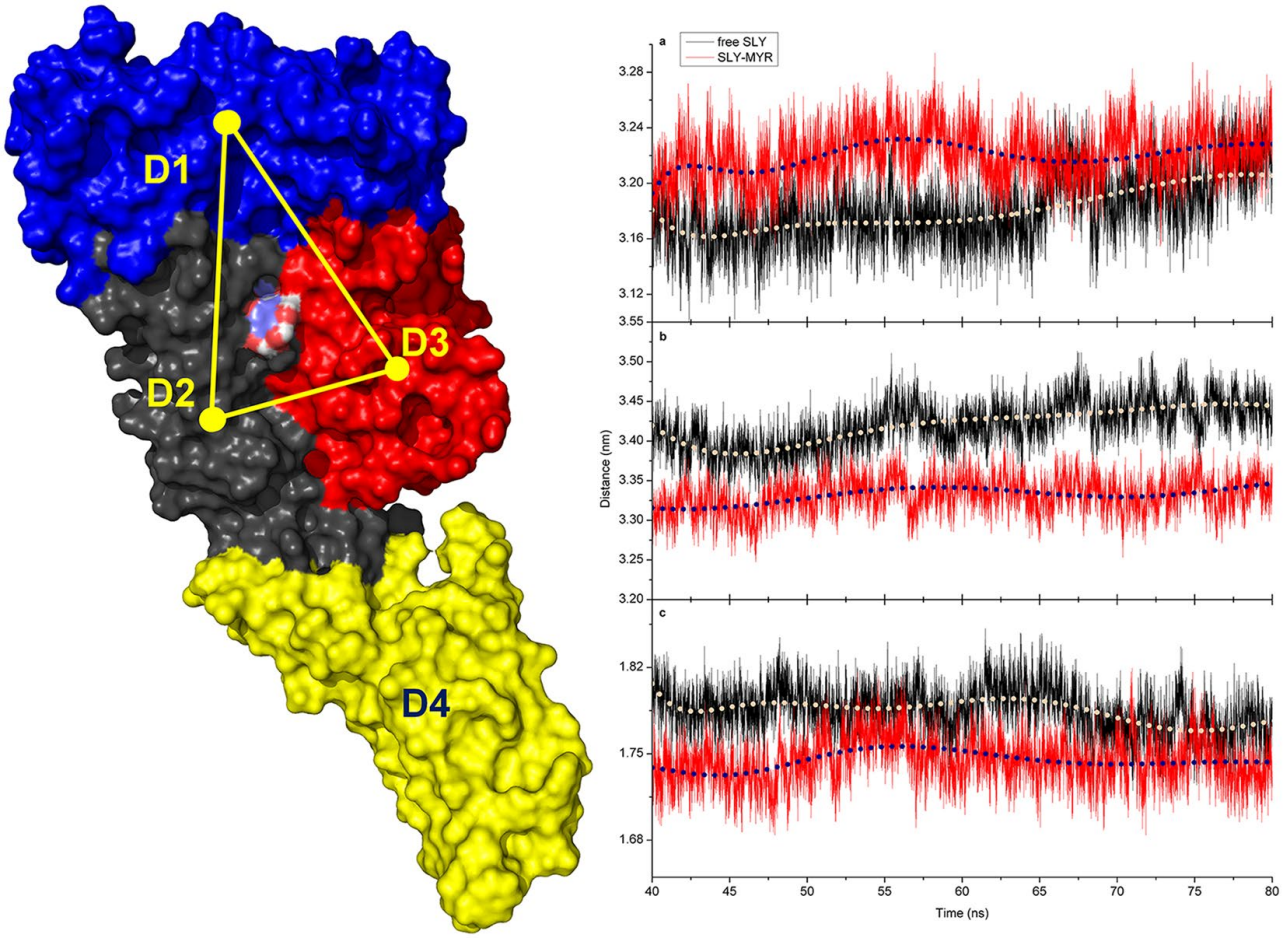
Conformational changes of SLY bound with MYR. The distances from D1 to D2 (**a**), D1 to D3 (**b**) and D2 to D3 (**c**) were calculated for the SLY-MYR complex system and the free SLY system.

According to previous reports, the conformational change of SLY should be complete to achieve haemolytic activity through monomeric oligomerization^7–9^. In the present study, the haemolytic activity of SLY was effectively suppressed by MYR, implying that the conformational change of SLY from a monomer to an oligomer was restricted as a result of the binding of SLY with MYR. Subsequently, PCA of the SLY-MYR complex system was performed to explore the key movements of SLY with or without MYR. As shown in Fig. 5a and b, obvious extended motion between D1 and D2 or 3 was observed in the first element (PC1) of the free SLY system. Moreover, in the second element (PC2) of the free SLY system, an approaching motion from D3 to D2 was evident. However, these two forms of movement were substantially weakened in PC1 and PC2 of the SLY-MYR complex system, as shown in Fig. 5c and d. The distances from D1 to D2, D1 to D3 and D2 to D3 were calculated for the SLY-MYR complex system and free SLY system, as shown in Fig. 6. The average distances from D1 to D2, D1 to D3 and D2 to D3 in the free SLY system were 3.18, 3.42 and 1.79 nm, respectively. However, in the complex system, the average distances from D1 to D2, D1 to D3 and D2 to D3 were 3.23, 3.33 and 1.73 nm, respectively, thus differing from those for free SLY. Therefore, the motion of D1, D2 and D3 in the monomeric SLY system was blocked as a result of the binding of MYR to the gap region between D2 and D3.

**Figure 7:**
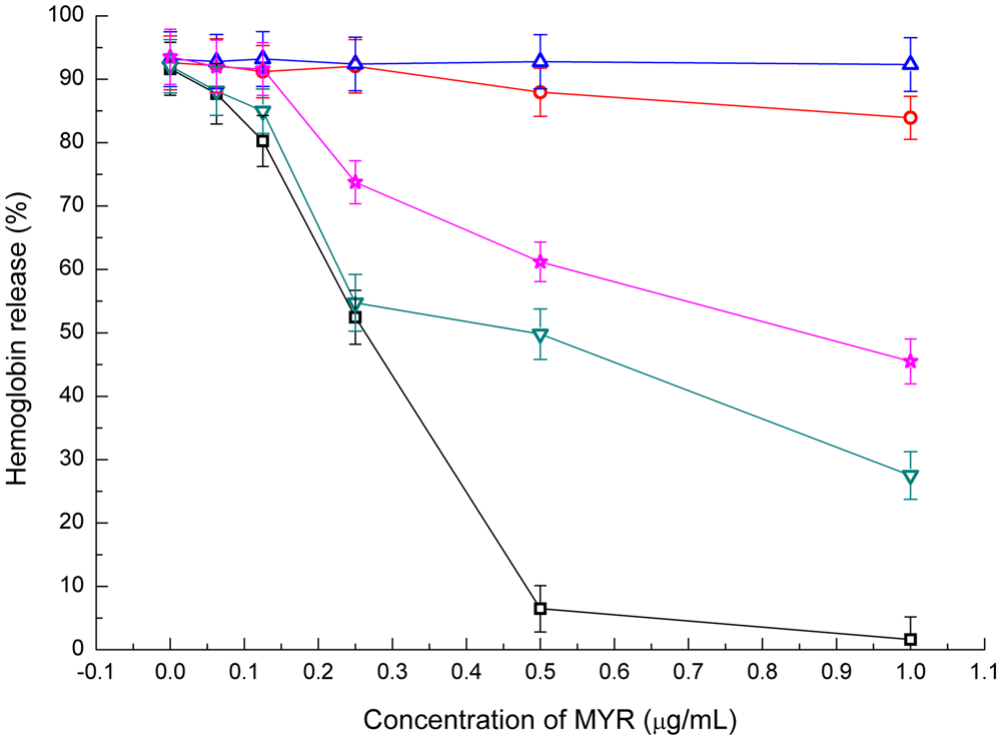
Results of haemolysis release assays performed with SLY. The haemolysis of WT-SLY (square) was reduced by the addition of MYR. However, the addition of MYR to N112A (circle), D179A (up-triangle) N82A (down-triangle) and S84A (star), did not result in inhibitory effects.

To verify this conclusion, haemolysis assays were performed for the complex systems of WT-SLY with MYR and the SLY mutants with MYR based on haemoglobin release from sheep red blood cells. Figure 7 shows that the haemolytic activity of SLY was obviously inhibited by the addition of 0.1 to 0.5 μg/mL MYR in a dose-dependent manner. Again, N82A, S84A, N112A and D179A showed high haemolytic activity compared with WT-SLY. However, MYR lost effective inhibitory activity for the mutants. These findings suggest that the haemolytic activity of SLY can be effectively reduced through the binding of MYR to the gap region between D2 and D3 in SLY.

Furthermore, the pseudo component of SLY sequence was analyzed by using the web server, Pse-in-One 2.0^25,26^. As shown in Figure S1, the results of the pseudo component were consistent with those of molecular modeling.

## Experimental Section

### Molecular modeling

The crystal structure of SLY obtained from the Protein Data Bank (PDB) and the PDB codes of 3HVN were employed as the initial coordinates for the molecular docking calculations using the Autodock 4.0 package^27–29^, and the Gaussian 03 programme was used to optimize the 3D structure of MYR at the B3LYP/6-31 G* level. The detailed docking procedure was performed as previously reported^30–32^. The 3D structure of SLY bound with MYR obtained through molecular docking was employed for the MD simulation. The interaction between SLY and MYR at the atomic level was investigated using the Gromacs 4.5.5 package for molecular modelling^33^. The detailed MD simulation was executed as previously reported^30–32^. Other computational methods are described in the Supplementary Materials.

## Conclusions

In the literature, molecular modelling has proven an effective, simple and inexpensive method for exploring the structure information of protein except for the precision accuracy to be improved with the development of computer hardware. Accordingly, in this study, the natural compound myricetin (MYR) can effectively suppress the haemolytic activity of SLY via directly binding to the gap region between domains 2 and 3 of SLY. Based on molecular dynamics simulations, mutational analysis and fluorescence-quenching assays, a novel inhibition mechanism was explored: the motion of D1, D2 and D3 in monomeric SLY was blocked through the binding of MYR to the gap region between D2 and D3, leading to loss of the haemolytic activity of SLY. These results are well consistent with those of web server, Pse-in-One 2.0^34^, based on the Kmer mode, which could contribute to the development of new and more effective antibacterial agents.

## Electronic supplementary material

Supplementary materials
