# Supplementary material for: Investigation of the inhibition effect and mechanism of myricetin to Suilysin by molecular modeling
Source: Sci Rep. 2017 Sep 18;7:11748. doi: 10.1038/s41598-017-12168-y (PMC5603505; doi:10.1038/s41598-017-12168-y)
Supplement: Supplementary file 1 — Supplementary materials [file 41598_2017_12168_MOESM1_ESM.pdf]

Investigation of the inhibition effect and mechanism of myricetin to Suilysin by molecular modelling

Xiaodi Niu, Lin Sun, Guizhen Wang, Yawen Gao, Yanan Yang, Xiyan Wang, Hongsu Wang*

College of food science and engineering, Jilin University

*Corresponding author

E-mail: wanghs@jlu.edu.cn (H. S. Wang)

Method and computation

Molecular dynamics simulation

All the simulations and the analysis of the trajectories were performed with Gromacs 4.5.5 software [1] using the Amber99sb force field and the TIP3P water model [2]. The SLY-MYR system was first energy-relaxed with 2000 steps of steepest-descent energy minimization followed by another 2000 steps of conjugate-gradient energy minimization. The system was then equilibrated by a 500 ps MD run with positional restraints on both the protein and ligand to allow relaxation of the solvent molecules. The first equilibration run was followed by a 100 ns MD run without position restraints on the solute. The first 30 ns of the trajectory were not used in the subsequent analysis to minimize convergence artefacts. Equilibration of the trajectory was checked by monitoring the equilibration of quantities, such as the root-mean-square deviation (RMSD) with respect to the initial structure, the internal protein energy, and fluctuations calculated for different time intervals. The electrostatic term was described with the particle mesh Ewald algorithm. The LINCS [3] algorithm was used to constrain all bond lengths. For the water molecules, the SETTLE algorithm [3] was used. A dielectric permittivity, $\varepsilon = 1$, and a time step of 2 fs were used. All atoms were given an initial velocity obtained from a Maxwellian distribution at the desired initial temperature of 300 K. The density of the system was adjusted during the first equilibration runs at NPT conditions by weak coupling to a bath of constant pressure ($P_0 = 1$ bar, coupling time $\tau_P = 0.5$ ps) [4]. In all simulations, the temperature was maintained close to the intended values by weak coupling to an external temperature bath with a coupling constant of 0.1 ps. The proteins and the rest of the system were coupled separately to the temperature bath. The structural cluster analysis was carried out using the method described by Daura and co-workers with a cutoff of 0.25 nm [4].

The MYR parameters were estimated with the antechamber program [5] and AM1-BCC partial atomic charges from the Amber suite [6]. Analysis of the trajectories was performed using VMD, PyMOL analysis tools and Gromacs analysis tools.

Calculation of the binding free energy

In this work, the binding free energies are calculated using MM-PBSA approach [7, 8] supplied with Amber 10 package. We choose a total number of 100 snapshots evenly from the last 10 ns on the MD trajectory with an interval of 10 ps. The MM-PBSA method can be conceptually summarized as:

$$\Delta G_{bind} = \Delta G_{complex} - [\Delta G_{protein} + \Delta G_{lig}] \quad 1$$

$$\Delta G_{bind} = \Delta H - T\Delta S \quad 2$$

where ΔH of the system is composed of the enthalpy changes in the gas phase upon complex formation (ΔE_{MM}) and the solvated free energy contribution (ΔG_{sol}), while $-T\Delta S$ refers to the entropy contribution to the binding. Eq. 2 can be then approximated as shown in Eq. 3:

$$\Delta G_{bind} = \Delta E_{MM} + \Delta G_{sol} - T\Delta S \quad 3$$

where ΔE_{MM} is the summation of the van der Waals (ΔE_{vdw}) and the electrostatic (ΔE_{ele}) interaction energies.

$$\Delta E_{MM} = \Delta E_{vdw} + \Delta E_{ele} \quad 4$$

In addition, ΔG_{sol}, which denotes the solvation free energy, can be computed as the summation of an electrostatic component ($\Delta G_{ele,sol}$) and a nonpolar component ($\Delta G_{nonpolar,sol}$), as shown in Eq. 5:

$$\Delta G_{sol} = \Delta G_{ele,sol} + \Delta G_{nonpolar,sol} \quad 5$$

The interactions between MYR and each residue of SLY are analyzed using the MM-PBSA decomposition process applied in the MM-PBSA module in Amber 10. The binding interaction of each ligand-residue pair includes three terms: the Van der Waals contribution (ΔE_{vdw}), the electrostatic contribution (ΔE_{ele}), and the solvation contribution (ΔE_{sol}). All energy components are calculated using the same snapshots as the free energy calculation.

Principal component analysis

Principle component analysis (PCA) [9, 10] was carried out to address the collective motions of SLY/SLY-MYR complex by using the positional covariance matrix C_α of the atomic coordinates and its eigenvectors. The elements of the

positional covariance matrix C_α are defined by eq 6:

$$C_i = \left\langle \left(x_i - \langle x_i \rangle\right)\left(x_j - \langle x_j \rangle\right) \right\rangle \left(i, j = 1, 2, 3, \ldots, 3N\right) \quad 6$$

where x_i is the Cartesian coordinate of the ith C_α atom, N is the number of C_α atoms considered, and $\langle x_i \rangle$ represents the time average over all of the configurations obtained in the simulation. The eigenvectors of the covariance matrix, V_k, obtained by solving $V_k^T CV_k = \lambda_k$, stand for a set of 3N-dimensional directions, or principal modes, along which the fluctuations observed in the simulation are uncoupled with respect to each other and can be analyzed separately.

In this work, to define the dominant motion over an MD simulation, the collective motions of the complex were addressed by using the positional covariance matrix, C_α, of the atomic coordinates and its eigenvectors based on PCA. The Gromacs 4.5.5 module was used to perform PCA, and the trajectories were obtained from the previous MD simulations.

Fluorescence-quenching assay

The binding constants (K_A) between WT-SLY, mutant SLY and MYR were measured using the fluorimetric quenching way. **[11-13]**

Site-Directed Mutagenesis

Three amino-acid mutations (N82A-SLY, S84A-SLY, N112A-SLY and D179A-SLY) were created using a QuikChange site-directed mutagenesis kit. The template plasmid was the pET28a-PLY plasmid noted above.

Haemolysis assay

The inhibitory effect of MYR on the hemolytic activity of the purified SLY was evaluated as previously described using defibrinated sheep red blood cells **[14]**. Briefly, 10 µL of purified SLY (0.4 µM) was added to 965 µL of PBS with various MYR concentrations (0, 20, 40 and 60 µM), and the mixtures were vortexed and incubated for 20 min at 37 °C. Then, 25 µL of defibrinated sheep red blood cells was added to the system, and the mixture was incubated for 10 min at 37 °C. After centrifugation at 3000g for 5 min, the hemolytic activity was determined by measuring the $OD_{543\ nm}$ of the supernatants.

The pseudo component analysis of SLY sequence

The pseudo component of SLY sequence was analyzed by using the web server, Pse-in-One 2.0. In the Pse-in-One web server, the Kmer mode was selected, with the parameter value of 3.

Reference:

1. Hess B, Kutzner C, Spoel, D. van der, Lindahl E. GROMACS 4: algorithms for highly efficient, load-balanced, and scalable molecular simulation. J Chem Theory Comput. 2008; 4: 435-447.
2. Jorgensen WL, Chandrasekhar J, Madura JD, Impey RW, Klein ML. Comparison of simple potential functions for simulating liquid water. J Chem Phys. 1983; 79: 926-935.
3. Ryckaert JP. Numerical integration of cartesian equations of motion of a system with constrained molecular dynamics of N-alkanes. J Chem Phys. 19997; 23: 327-341.
4. Berendsen HJC, Postma JPM, Gunsteren WF van, Dinola A, Haak JR. Molecular dynamics with coupling to an external bath. J Chem Phys. 1984; 81: 3684-3690.
5. Wang J, Wang W, Kollman PA, Case DA. Automatic atom type and bond type perception in molecular mechanical calculations. J Mol Graph Model 2006; 2: 247-260.
6. Jakalian A, Jack DB, Bayly CI. Fast, efficient generation of high-quality atomic charges. AM1-BCC model: II. Parameterization and validation. J Comput Chem. 2002; 23: 1623-1641.
7. Punkvang A, Saparpakorn P, Hannongbua S, Wolschann P, Beyer A, Pungpo P. Investigating the structural basis of arylamides to improve potency against M. tuberculosis strain through molecular dynamics simulations. Eur J Med Chem. 2010; 45: 5585-5593.
8. Schaffner-Barbero C, Gil-Redondo R, Ruiz-Avila LB, Huecas S, Lappchen T, den Blaauwen T, Diaz JF, Morreale A, Andreu JM. Insights into Nucleotide Recognition by Cell Division Protein FtsZ from amant-GTP Competition Assay

and Molecular Dynamics. Biochemistry. 2010; 49: 10458-10472.

9. Amadei A, Linssen AB, Berendsen HJ. Essential Dynamics of Proteins. Proteins. 1993; 17: 412-425.
10. Du Y, Yang HY, Xu YH, Cang XH, Luo C, Mao YY, Wang YY, Qin GG, Luo XM, Jiang HL. Conformational Transition and Energy Landscape of ErbB4 Activated by Neuregulin1β: One Microsecond Molecular Dynamics Simulations. J Am Chem Soc. 2012; 134: 6720-6731.
11. Dong J, Qiu JZ, Zhang Y, Lu CJ, Dai XH, Wang JF, Li HE, Wang X, Tan W, Luo MJ, Niu XD, Deng XM. Oroxylin A Inhibits Hemolysis via Hindering the Self-Assembly of α-Hemolysin Heptameric Transmembrane Pore. PLOS Computational Biology. 2013; 9: e100286.
12. Niu XD, Qiu JZ, Wang X, Gao XH, Dong J, Wang JF, Li HE, Zhang Y, Dai XH, Lu CJ, Deng XM. Molecular insight into the inhibition mechanism of cyrtominetin to α-hemolysin by molecular dynamics simulation. Eur J Med Chem. 2013; 62: 320-328.
13. Qiu JZ, Wang DC, Zhang Y, Dong J, Wang JF, Niu XD. Molecular Modeling Reveals the Novel Inhibition Mechanism and Binding Mode of Three Natural Compounds to Staphylococcal α-Hemolysin. PLOS ONE. 2013; 8: e80197.
14. Li HE, Zhao XR, Wang JF, Dong Y, Meng S, Li R, Niu XD, Deng XM. β-sitosterol interacts with pneumolysin to prevent Streptococcus pneumonia infection. Scientific Reports. 2015; 5: 17668.

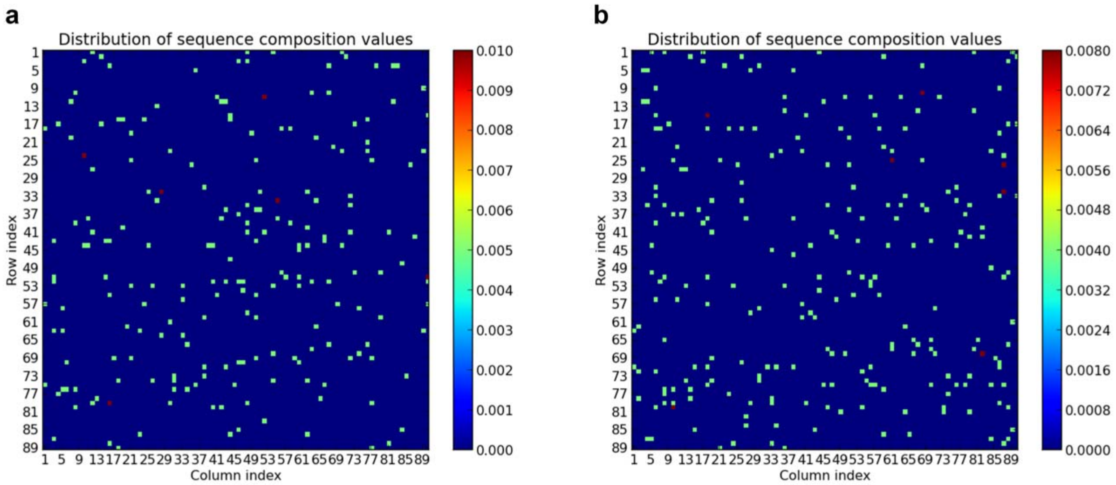

Figure S1. The distribution of sequence composition values of SLY. (a) residues from 32-241; (b) residues from 242-499.
